# Expression of a Finger Millet Transcription Factor, *EcNAC1,* in Tobacco Confers Abiotic Stress-Tolerance

**DOI:** 10.1371/journal.pone.0040397

**Published:** 2012-07-11

**Authors:** Venkategowda Ramegowda, Muthappa Senthil-Kumar, Karaba N. Nataraja, Malireddy K. Reddy, Kirankumar S. Mysore, Makarla Udayakumar

**Affiliations:** 1 Department of Crop Physiology, University of Agricultural Sciences, Bangalore, Karnataka, India; 2 Plant Biology Division, The Samuel Roberts Noble Foundation, Ardmore, Oklahoma, United States of America; 3 International Centre for Genetic Engineering and Biotechnology, New Delhi, Delhi, India; National Taiwan University, Taiwan

## Abstract

NAC (NAM, ATAF1-2, and CUC2) proteins constitute one of the largest families of plant-specific transcription factors and have been shown to be involved in diverse plant processes including plant growth, development, and stress-tolerance. In this study, a stress-responsive *NAC* gene, *EcNAC1*, was isolated from the subtracted stress cDNA library generated from a drought adapted crop, finger millet, and characterized for its role in stress-tolerance. The expression analysis showed that *EcNAC1* was highly induced during water-deficit and salt stress. *EcNAC1* shares high amino acid similarity with rice genes that have been phylogenetically classified into stress-related *NAC* genes. Our results demonstrated that tobacco transgenic plants expressing *EcNAC1* exhibit tolerance to various abiotic stresses like simulated osmotic stress, by polyethylene glycol (PEG) and mannitol, and salinity stress. The transgenic plants also showed enhanced tolerance to methyl-viologen (MV) induced oxidative stress. Reduced levels of reactive oxygen species (ROS) and ROS-induced damage were noticed in pot grown transgenic lines under water-deficit and natural high light conditions. Root growth under stress and recovery growth after stress alleviation was more in transgenic plants. Many stress-responsive genes were found to be up-regulated in transgenic lines expressing *EcNAC1.* Our results suggest that *EcNAC1* overexpression confers tolerance against abiotic stress in susceptible species, tobacco.

## Introduction

Plants maintain cellular homeostasis under abiotic stress by adjusting their metabolic systems. The early events of plant adaptation to the environmental stresses involve stress-signal perception and transduction leading to the expression of stress-responsive genes and activation of various physiological and metabolic responses [1], [2], [3]. The products of stress-responsive genes can either directly protect cells against abiotic stress or regulate the expression of other genes. The stress regulatory molecules, including mitogen-activated protein kinases, histidine kinases, Ca^2+^-dependent protein kinases, as well as transcription factors are well characterized [4], [5]. The expression of stress-responsive genes is largely governed by specific transcription factors. Stress-responsive transcription factors predominantly interact with promoter elements of stress genes often resulting in expression of many functional genes. This has been elegantly shown for DREB and bZIP transcription factors which recognize drought responsible element (DRE) and the abscisic acid responsive element (ABRE), respectively, and activate an array of stress genes [6]. Several families of transcription factors, including ERF/AP2, HSF, bZIP, MYB, MYC, NAC, WRKY, and Zinc-finger, have been well characterized for their regulatory roles in eliciting plant stress-responses [5], [7]–[10].

NAC family proteins are plant-specific transcription factors that are characterized by a conserved DNA-binding NAC domain located in the N-terminal region and a variable C-terminal domain [11]. Although the genomes of rice and *Arabidopsis* were predicted to contain 140 and 75 *NAC* genes, respectively, [12], [13], only a few members of this large family have been characterized and most of them were reported to be involved in plant growth, development, and hormone signaling [14]. Recently, a few NAC proteins have been described as mediators of biotic and abiotic stress signaling. Expression analysis showed that *NAC* genes, such as *ANAC019, ANAC055, and ANAC072* from *Arabidopsis* [15], and *BnNAC* from *Brassica napus* [16], were induced by various environmental stresses. Furthermore, overexpression of a few *NAC* genes has been shown to improve abiotic stress-tolerance in *Arabidopsis* and rice through activation of several stress genes [17]–[21]. *NAC* genes seem to have non-redundant functions though they are stress-induced [17], [18], [22]. In addition, the function of same transcription factor could behave differently between the species. Surprisingly, overexpression of seven stress-induced *NACs* did not give tolerant phenotype in *Arabidopsis* [23]. However, Dubouzet et al. [24] demonstrated that overexpression of *OsDREB1A* in *Arabidopsis* and rice improves tolerance to drought, salinity and freezing although a few target genes were not up-regulated in *Arabidopsis,* which were induced in rice.

Significant genetic diversity exists among plant species for stress adaptation; hence elucidating the transcriptome from abiotic stress adapted species has greater significance. Several reports have shown that genes from stress adapted species are functionally more efficient in imparting tolerance [25], [26], [27]. Although the studies with *Arabidopsis* dissected several abiotic stress pathways, *Arabidopsis* being a susceptible species the focus has now been on its related tolerant family or species, namely, *Thellungiella halophila, Thlaspi caerulescens* and *Arabidopsis halleri* [28]–[31]. Apart from these, other stress adapted species and resurrection plants with very high tolerance threshold may possess mechanisms and genes which make them survive extreme conditions. Two of our recent studies [32], [33] and also other reports [34], [35] support this concept. Though stress adapted crops such as sorghum and maize genomes have several *NAC* genes, there is no information on the functional relevance of these genes in imparting stress-tolerance and stress-responsive *NACs* are yet to be identified in these species. Hence, discovery and characterization of stress-responsive NAC transcription factors from stress adapted species would provide insight on the importance of these transcription factors.

Figner millet [*Eleusine coracana* (L.) Gaertn], a crop which is widely grown in semi-arid and arid tropics of Africa and Asia is widely adapted to a broad range of abiotic stresses. Finger millet survives under severe water-deficit and osmotic stress and shows remarkable recovery on alleviation of stress [33], [36]. In this study, we report the identification of a putative NAC family transcription factor, denoted as *EcNAC1*, from a subtracted cDNA library of finger millet. We show that *EcNAC1* is induced by various abiotic stresses and expression of this gene in susceptible species, tobacco, imparts abiotic stress-tolerance.

## Materials and Methods

### Construction of cDNA Library in Finger Millet and Identification of *EcNAC1*

#### Plant material and growth conditions

Finger millet (var. GPU28) seeds were sown in pots filled with mixture of soil, sand and farmyard manure (3∶1∶1 proportion). Seeds of *Nicotiana tabacum* [tobacco (var. KST 19)] were germinated, transplanted in soil at two or three leaf stage, and maintained in greenhouse conditions at a day/night temperature of 25±1°C.

#### Stress imposition

Twenty-five-days after sowing, water-deficit stress was imposed to finger millet seedlings simulating field condition. Rain-out shelter was used to protect plants from adverse weather; otherwise plants were exposed to natural vapor pressure deficit. Desired stress levels were achieved gradually by gravimetric approach [37], [38]. Progressive stress was applied for a set of plants over a period of 5 days to reach 80% field capacity (FC), likewise 8 days to reach 60% and 10 days to reach 35% FC. Leaf tissue was harvested on same day for all stress levels. To study the expression of native *NAC* gene in tobacco, dehydration stress was created on two weeks old seedlings by transferring them to Petri-plates (9 cm diameter) containing MS medium supplemented with 5% PEG-10000 (w/v) and tissue was harvested at 24 h after treatment. To create salinity stress, two weeks old seedlings were watered with 200 mM NaCl solution and tissue was harvested at 24 h. In all the treatments a set of control seedlings were maintained.

#### cDNA library

Total RNA was isolated [39] from fully expanded young leaves of both control and stressed (80%, 60% and 35% FC) finger millet plants and subtractive hybridization was performed according to Mishra et al. [40]. The differentially expressed subtracted poly(A+) RNA was used to synthesize double-stranded cDNA and uni-directionally ligated to a phage lambda-ZAP vector, *in-vitro* packaged and infected to XL1-blue *Escherichia coli* cells according to the manufacturer’s instructions using a Uni-ZAP XR cDNA library construction kit (Stratagene, CA, USA). The cDNA inserts from individual recombinant plaques were verified by PCR with universal M13 forward and reverse primers using 1 µl of recombinant phage suspension as a template in 50 µl reaction volume for 30 cycles with the following PCR conditions, 94°C for 1 min, 55°C for 1 min and 72°C for 2 min. PCR amplified cDNA inserts were sequenced using universal T7 primers.

#### Identification of *EcNAC1*

A truncated NAC domain containing gene was identified from the sequenced clones of cDNA library by NCBI BLAST search and comparative analysis. The missing 5′ end was amplified using RNA Ligase Mediated Rapid Amplification of cDNA Ends (RLM-RACE) kit (Invitrogen Corporation, Carlsbad, CA, USA) according to manufacturer’s instruction.

#### Bioinformatic analysis

The *EcNAC1* was compared with 140 *NAC’s* of rice [12]. The full-length protein sequences were multiple aligned with deduced protein sequence of *EcNAC1* and phylogenetic tree was constructed with the program ClustalW [41]. The program multiple EM for motif elicitation (MEME) [42] was used to predict the potential motifs in the sequences. All motifs discovered by MEME were searched in the InterPro database with InterProScan [43]. PSORT [44] and NetNES [45] tools were used to predict the nuclear localization and nuclear export signals, respectively.

### Construction of Plant Expression Vectors and Plant Transformation

#### Constructs

Coding region of *EcNAC1* was amplified using cDNA synthesized from finger millet leaf tissue, undergoing water-deficit stress, with specific primers (Table S1A). The expression cassettes, *CaMV35S:EcNAC1:NOS* and *4xABRE:EcNAC1:NOS* (*4xABRE* is a synthetic promoter with *CaMV35S* minimal promoter), were inserted into binary vector *pGreen0179* [46] containing a hygromycin resistance selectable marker. The binary vector *pGreen0179* with *EcNAC1* expression cassettes and *pSoup* plasmids were co-mobilized into *Agrobacterium tumefaciens* strain LBA4404.

#### Transformation

*In-vitro* regeneration approach was followed to generate transgenic tobacco plants [47]. Leaf discs from healthy tobacco plants (var. KST 19) were co-cultivated for 15 min with *A. tumefaciens* harbouring the recombinant binary vectors and cultured on Murashige and Skoog (MS) [48] agar medium supplemented with 6-benzylaminopurine (2.25 mg/l), naphthalene acetic acid (0.1 mg/l), cefotaxime (400 mg/l), and hygromycin (30 mg/l). The regenerated shoots were rooted on MS basal medium containing hygromycin (30 mg/l). Profusely rooted plantlets were transferred into pots and maintained in greenhouse following acclimatization for two weeks.

### Molecular Analysis of Tobacco Transgenic Plants

#### Gene expression by RT-PCR and quantitative RT-PCR

First-strand cDNA was synthesized from total RNA (2 µg) primed by oligo (dT)_15_ using Moloney Murine Leukemia Virus Reverse Transcriptase (MMLV-RT; MBI Fermentas, Hanover, MD, USA) according to the manufacturer’s instructions. For expression analysis, cDNA pool was used as a template. Thirty cycles of PCR (with 3 min of initial denaturation at 95°C, 94°C for 1 min, 50–58°C for 1 min, 72°C for 1 min) amplification, with a final extension at 72°C for 5 min was performed. *EcNAC1* was amplified with the primers specific to the 284 bp 3′ terminal region of its open reading frame (ORF) (Table S1B). To study the expression of target genes of *EcNAC1*, RT-PCR was performed with 14 closest tobacco homologs of known *SNAC1* target genes [17] (Table S1C & Table S2). House-keeping gene, either *Actin* or *Elongation factor 1α,* was used in all expression studies and treated as control. The RT-PCR products were loaded on a 1% agarose gel and bands were analysed using ImageJ software version 1.34 s (National Institutes of Health) to calculate the changes in relative density of bands keeping wild-type as one. To study the expression pattern of *EcNAC1* under water-deficit stress and salinity, quantitative RT-PCR (qRT-PCR; Opticon 2, MJ research, USA) was performed using the fluorescent dye SYBR-Green (DyNAmo SYBR-Green qRT-PCR kit, Finnzymes, Finland) for 30 cycles following the manufacturer’s protocol. The relative expression levels under a given stress condition compared to its corresponding control condition was calculated using Relative Expression Software Tool (REST) [49]. The data was normalized to expression levels of *Actin*. Similarly, the expression pattern of *NtNAC* was studied under dehydration and salinity using specific primers (Table S1D) and the data was normalized to expression levels of *Elongation factor 1α*.

### Abiotic Stress-tolerance of *EcNAC1* Transgenic Tobacco Plants

#### Abiotic stress treatment at seedling stage

Transgenic tobacco (T_1_) plants expressing *EcNAC1* (constitutive transgenic lines - S1, S3, S4, S7 and S8; and stress-inducible transgenic lines - A1, A2, A4, A5 and A6) were evaluated under various stress conditions. For growth analysis, 15-day-old seedlings selected on 30 mg/l hygromycin were transferred to Petri-plates (9 cm diameter) containing MS medium supplemented with 5% PEG-10000 (w/v), and 5 µM MV, separately, and fresh weight was recorded. For examining root growth, 15-day-old seedlings were grown in Petri-plates (12 cm diameter) containing MS +200 mM NaCl. For long-term stress treatment, 15-day-old seedlings were transferred to culture bottles containing MS medium supplemented with various concentrations of mannitol (100, 200 and 300 mM), and NaCl (100, 200 and 300 mM) and performance was measured after 30-days.

#### Imposition of water-deficit

For water-deficit stress treatment under greenhouse conditions, 15-day-old seedlings were transferred to pots filled with potting mixture of known weight. After seedling establishment, soil water status was monitored by gravimetric approach [37], [38] and soil FC was gradually brought down to 35% and maintained for 10-days. Relative humidity in the greenhouse ranged from 75–80% and temperature was maintained around 29–30°C during day time and 24–26°C during the night period. Stress effect was assessed at the end of stress period by quantifying ROS and malondialdehyde (MDA) content in leaf tissue. After re-watering the recovery response was studied after 15-days by measuring total biomass accumulated.

#### Determination of superoxide radical (O._2_^−^)

Leaf discs were incubated in 1 ml of K-phosphate buffer (20 mM, pH 6.0) containing 500 µM XTT (Polysciences Europe, Eppelheim, Germany) in darkness at 25°C on a shaker. The increase in absorbance (A_470_) in the incubation medium was measured using spectrophotometer [50].

#### Determination of hydrogen peroxide (H_2_O_2_)

Leaf discs were pre-incubated for 30 min in 3 ml of K-phosphate buffer (20 mM, pH 6.0) to remove pre-formed H_2_O_2_ and were then incubated in 3 ml of the same buffer containing 5 µM scopoletin [7- hydroxy-6-methoxy-2H-1-benzopyran-2-one (Sigma Aldrich, St. Louis, MO USA)] [51] and 3 µg/ml horseradish peroxidase in darkness at 25°C on a shaker. The decrease in fluorescence (excitation: 346 nm, emission: 455 nm) in the incubation medium was measured using reagent blanks as reference.

#### Determination of hydroxyl radical (.OH)

Leaf discs were incubated in 1.5 ml of buffer containing 20 mM 2-deoxy-D-ribose (Sigma Aldrich, St. Louis, MO USA). The formation of the breakdown product MDA was determined by mixing 0.5 ml of centrifuged incubation medium with 0.5 ml of 2-thiobarbituric acid (10 g/l in 50 mM NaOH) and 0.5 ml of trichloroacetic acid (28 g/l). After heating in boiling water for exactly 10 min, cooling in tap water, and clarifying by centrifugation, the reaction product was measured fluorometrically (excitation: 532 nm, emission: 553 nm) against reagent blanks [52].

#### Estimation of MDA content

MDA content was quantified by thiobarbituric acid reactive substances assay [53] (Heath and Packer 1968). About 0.5–1 g of tissue was homogenized in 5 ml of 5% (w/v) trichloroacetic acid and the homogenate was centrifuged at 12000 g for 15 min at room temperature. The supernatant was mixed with an equal volume of thiobarbituric acid [0.5% in 20% (w/v) trichloroacetic acid], and the mixture was boiled for 25 min at 100°C, followed by centrifugation for 5 min at 7500 g to clarify the solution. Absorbance of the supernatant was measured at 532 nm and corrected for non-specific turbidity by subtracting the A_600_. MDA equivalents were calculated by the extinction coefficient of 155 M^−1 ^cm^−1^. Values of MDA contents were taken from measurements of three independent samples.

## Results

### Isolation of Water-deficit Induced Finger Millet *NAC* gene, *EcNAC1*

To monitor the changes in expression profiles of transcripts in response to dehydration, finger millet plants were exposed to different levels of gradual water-deficit stress and maintained at different FC viz., 100%, 80%, 60% and 35% (Figure S1). cDNA library was constructed from the leaf tissue following subtraction between the stress-exposed pooled samples and control-samples. The mRNA populations from stress-exposed seedlings were mixed with approximately five-fold excess of complimentary first-strand cDNA from corresponding control seedlings. Double-stranded cDNA synthesized from the subtracted poly(A+) RNA was used in generating the library. The cDNA inserts of selected recombinant plaques were successfully amplified by PCR using universal M13 primers and used for sequencing. One of the cDNA clones showed maximum homology to a gene encoding a putative NAC domain containing protein. The identified cDNA was approximately 1 kb with an ORF that could encode a protein of 246 amino acids without the starting methionine suggesting that the *EcNAC1* cDNA clone was truncated in the 5′ region. The 3′ end seemed to be intact with 215 bp 3′ un-translated region followed by a poly(A+) tail. In order to identify and isolate the missing 5′ end of *EcNAC1* cDNA, we performed 5′ RACE-PCR with gene specific primer (CAGTCATCCAACCTTAGCGATC) in the antisense orientation using RLM-RACE kit and amplified a fragment of 690 bp, which was cloned and sequenced. The RACE product shared an expected 145 bp homology with the partial *EcNAC1* cDNA clone as the gene specific primer used in the 5′ RACE was designed to amplify a 145 bp overlap at the 5′ end of the partial cDNA clone. This observation confirmed that the fragment of 690 bp was indeed a part of *EcNAC1* cDNA. As revealed, total size of *EcNAC1* cDNA clone was 1547 bp (GenBank accession EU439937) with an ORF of 1110 bp, encoding 369 amino acid polypeptide flanked by 186 and 215 bp 5′ and 3′ un-translated regions, respectively.

### Bioinformatic Analysis of *EcNAC1*

**Figure 1:**
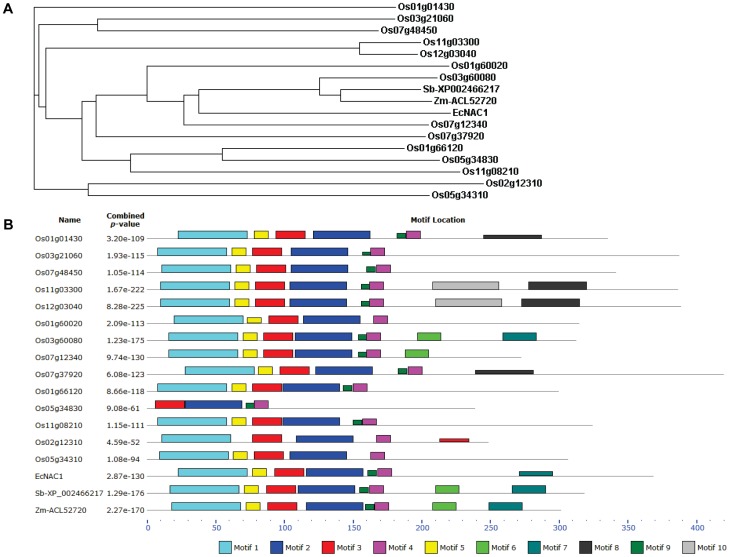
Bioinformatic analysis of NAC protein sequences. (A) Phylogram was derived for rice, sorghum and maize NAC protein sequences which clustered with EcNAC1 using the program ClustalW. (B) Prediction of putative motifs shared among these protein sequences using MEME program. Numbered boxes represent different putative motifs (annotations of these motifs in EcNAC1 are listed in Table S3).

Sequence homology search performed using the deduced amino acid sequence of *EcNAC1* against translated non-redundant nucleotide database using tBLASTN and protein database using BLASTP showed an overall 57–59% sequence identity to sorghum (GenBank ID: XP002466217), maize (GenBank ID: ACL52720) and rice (Os03g60080) genes as well as many other plant genes encoding NAC proteins. The NAC domain containing proteins are highly conserved across all plants and encoded by multi-gene family. The gene duplications and their subsequent divergence were central to multiplicity of the NAC family. It was very difficult to establish the evolutionary relationship of *EcNAC1* isolated in this study with other plant orthologs based on its NAC domain mediated trans-activation of several downstream genes. The availability of complete genome sequence of rice has provided a reference platform to identify the complete list of genes that encode NAC proteins in rice plants and allowed us to conduct phylogenetic analysis and establish the EcNAC1 relationship with other rice orthologs. A phylogenetic study was conducted using known 140 genes of NAC family full-length protein sequences from rice and also closest accessions from sorghum (GenBank ID: XP002466217) and maize (GenBank ID: ACL52720) along with EcNAC1 sequence by ClustalW program. The EcNAC1 was phylogenetically aligned to a cluster of 14 rice and also sorghum and maize NAC proteins and, particularly, evolutionarily very close to rice NAC ortholog Os03g60080 (SNAC1) (Figure 1A). In addition to the amino acid sequence homology, several structural motifs predicted based on the MEME program in EcNAC1 polypeptide are well conserved with the same order as in rice (Os03g60080) as well as sorghum and maize NAC proteins except for the motif 6 which is absent in EcNAC1, signifying the unique C-terminal region of the gene and also suggesting that these NAC proteins are evolutionarily evolved from a common ancestor (Figure 1B & Table S3). Amino acid sequence similarity of EcNAC1 with known stress-responsive NAC family genes suggests the plausible involvement of this gene in stress adaptation. When translated amino acid sequence was analyzed by PSORT for nuclear localization and nuclear export signals, EcNAC1 showed four residue pattern composed of three basic amino acids (RRR) and H (one residue at 190 and another at 122) with nuclear targeting certainty score of 0.98 for 1. Apart from that, the sequence also showed 7 residue pattern of nuclear targeting at position 176 with two basic residues, ten residues spacer and another basic region consisting of three basic residues out of five residues (KK NEWEKMQMKK GYRRR). In addition, NetNES tool predicted the residue “LAL” at 213 amino acid with calculated nuclear export signal score exceeding the threshold score suggesting the particular residue is expected to participate in a nuclear export signal. A comprehensive analysis of NAC family genes in rice and *Arabidopsis* (75 and 105 predicted NAC proteins, respectively) predicted conserved DNA binding domain at the N-terminal region and a variable C-terminal region which is known to be involved in transcriptional activation [11]. Analysis of EcNAC1 amino acid sequence using InterProScan predicted a NAC DNA binding domain from 24–176 amino acid. A conserved motif detected in the C-terminal region using the MEME program was also found in the SNAC1 [17]. It has been clearly shown that this C-terminal activation domain in SNAC1 had transactivation activity suggesting that EcNAC1 is also capable of transactivation.

### Expression Pattern of *EcNAC1* gene in Response to Abiotic Stress

**Figure 2:**
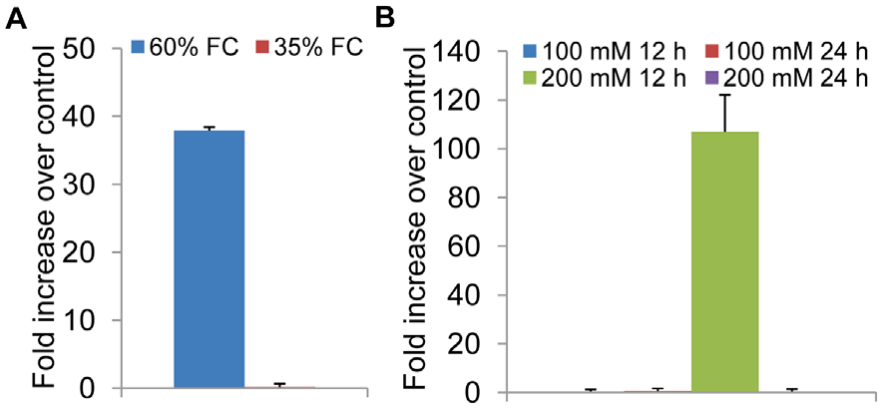
Quantitative expression of *EcNAC1* gene in response to water-deficit and salt stress. (A) Adapting a gradual stress imposition protocol, 25-day-old finger millet seedlings were subjected to water-deficit stress following gravimetric approach. (B) NaCl treatment was given to seedlings 3-days after germination. Accumulation of *EcNAC1* transcripts was determined by qRT-PCR with 2 µg of total RNA. The *Actin* gene was used as normalizer.

In plants, a multi-gene family encodes NAC domain containing proteins, with a very high sequence homology among the family members. Each of these genes responds differentially to environmental and/or developmental cues. Therefore, it is very difficult to monitor the expression of these genes individually by northern blot analysis due to cross hybridization. Therefore the relative expression of *EcNAC1* gene was monitored in finger millet in response to various stresses in comparison to non-stressed control seedlings by quantitative RT-PCR analysis using gene-specific primers. The mRNA purified from the stress exposed finger millet seedlings and their corresponding control seedlings was reverse transcribed separately into first-strand cDNA and used as a template. The *EcNAC1* transcript was detectable in both stress exposed and control seedlings. The up-regulation of *EcNAC1* gene in response to different environmental stresses is shown in Figure 2. In an attempt to capture both rapid and long-term changes in *EcNAC1* gene expression, we sampled the finger millet seedlings 12 and 24 h after exposure to NaCl. Under water-deficit stress the expression was seen at mild (60% FC) as well as severe stress (35% FC). The relative up-regulation of *EcNAC1* was ∼40 folds higher in response to mild water-deficit stress and down-regulated at severe water-deficit stress (Figure 2A). The relative up-regulation of *EcNAC1* gene was observed not only in response to mild water-deficit stress but also in response to salinity (200 mM NaCl at 12 h) stress with ∼110 fold increase (Figure 2B).

### Molecular Analysis of Transgenic Tobacco

The NAC transcription factor-mediated transcriptional activation of stress-responsive genes and their associated signaling networks regulate dynamic co-expression of several stress-responsive genes together to alleviate stress-induced cellular damage. To evaluate whether the ectopic transgenic overexpression of *EcNAC1* confers any stress-tolerance, we expressed the full-length *EcNAC1* cDNA cloned under the control of *CaMV35S* promoter as well as *4xABRE* stress-inducible promoter in a heterologous plant system, tobacco a relatively susceptible species. *4xABRE* is a synthetic promoter containing four copies of ABRE elements and is known to be induced by dehydration, high salinity, low temperature and ABA [54]. Both constitutive and stress-inducible constructs were transformed to tobacco following *A. tumefaciens*-mediated transformation. The putative transgenic tobacco plants were regenerated and selected on hygromycin containing medium. These plants were further screened for transgene integration by PCR (Figure S2, S3 & Table S1E). The PCR positive ten independent T_0_ plants (five each from constitutively expressing *EcNAC1* transgenic lines - S1, S3, S4, S7 and S8; and stress-inducible *EcNAC1* transgenic lines - A1, A2, A4, A5 and A6) were selfed individually and seeds were harvested for further analysis. Approximately 70–80% of seeds from these selected putative T_0_ transgenic lines germinated and developed into T_1_ seedlings on MS medium supplemented with hygromycin (30 mg/l) suggesting a 3∶1 ratio of Mendelian segregation. The RT-PCR analysis of stressed leaf tissue from transgenic plants confirmed the transgene expression (Figure S4A). Expression of the gene was not observed in wild-type tobacco plants, as there was no homology found in tobacco for the selected primers used for RT-PCR analysis. The relative density of the bands was determined to show the increase in transcript levels over wild-type (Figure S4B). Transgenic overexpression of *EcNAC1* under the constitutive *CaMV35S* promoter as well as *4xABRE* promoter did not show any phenotypic abnormities either during stress or non-stress growth conditions.

### Tolerance of *EcNAC1* Transgenic Tobacco to Osmotic Stress

**Figure 3:**
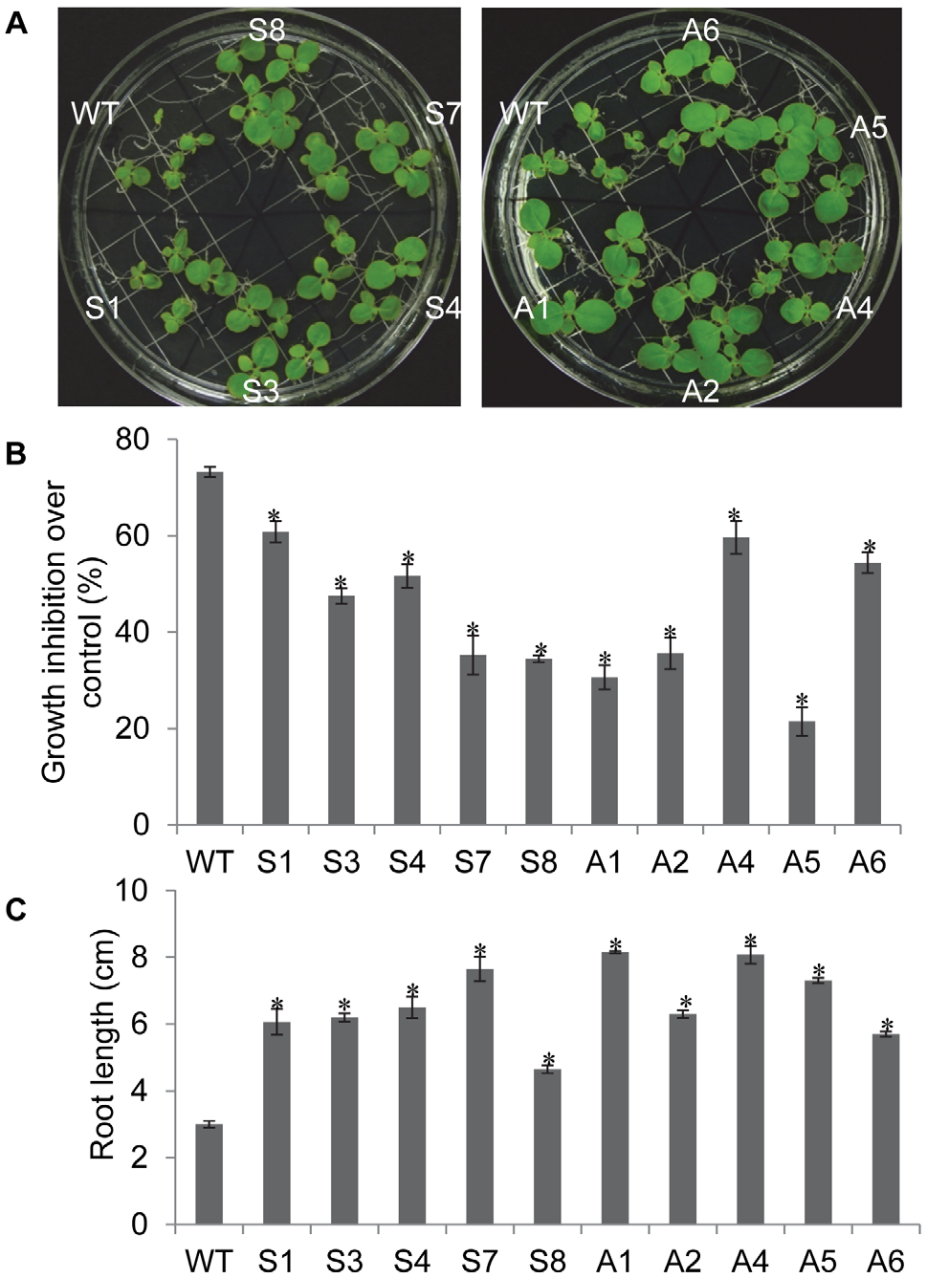
Short-term osmotic stress response of *EcNAC1* transgenic tobacco plants. 15-day-old T_1_ transgenic seedlings selected on hygromycin were inter-planted with wild-type seedlings on MS medium supplemented with 5% PEG and observations were taken after seven days of stress treatment. (A) Phenotype, (B) Reduction in fresh weights over control, and (C) Root elongation of transgenic tobacco plants under stress. Each bar value represents the mean ± sd (n = 12) of triplicate experiments (student’s t test; *P<0.05 versus wild-type).

In all the selected transgenic lines short-term stress response was studied by exposing to stress for seven days. Whereas long-term stress experiments by exposing to stress up to 30 days was carried out only in a few lines, S4, S7, A4 and A7, which showed superior stress-tolerance in short-term experiments. During short-term osmotic stress response studies, all the transgenic seedlings exposed to 5% PEG maintained better growth than wild-type as indicated by significantly lower growth inhibition (Figure 3A & B). Transgenic lines showed 1.5–3 fold less reduction in fresh weight compared to wild-type plants. The root growth was also better in transgenic plants under stress (Figure 3C). Under long-term stress created by exposure to mannitol, transgenic plants showed less reduction in fresh weight at higher concentration (300 mM) when compared to wild-type plants (Figure S5A & S5B). In addition, a densely developed root system was observed in transgenic plants. The root mass was 2–3 folds more in transgenic plants when compared to wild-type roots (Figure S5C). Wild-type plants were dwarf and the leaves were pale green, however transgenic plants maintained the greenness. Thus, these results clearly demonstrate that *EcNAC1* transgenic tobacco plants exhibit a significant level of tolerance to PEG and mannitol-induced osmotic stress.

### *EcNAC1* Transgenic Tobacco Plants Showed Rapid Root Elongation and Improved Growth Rate during Salt Stress

**Figure 4:**
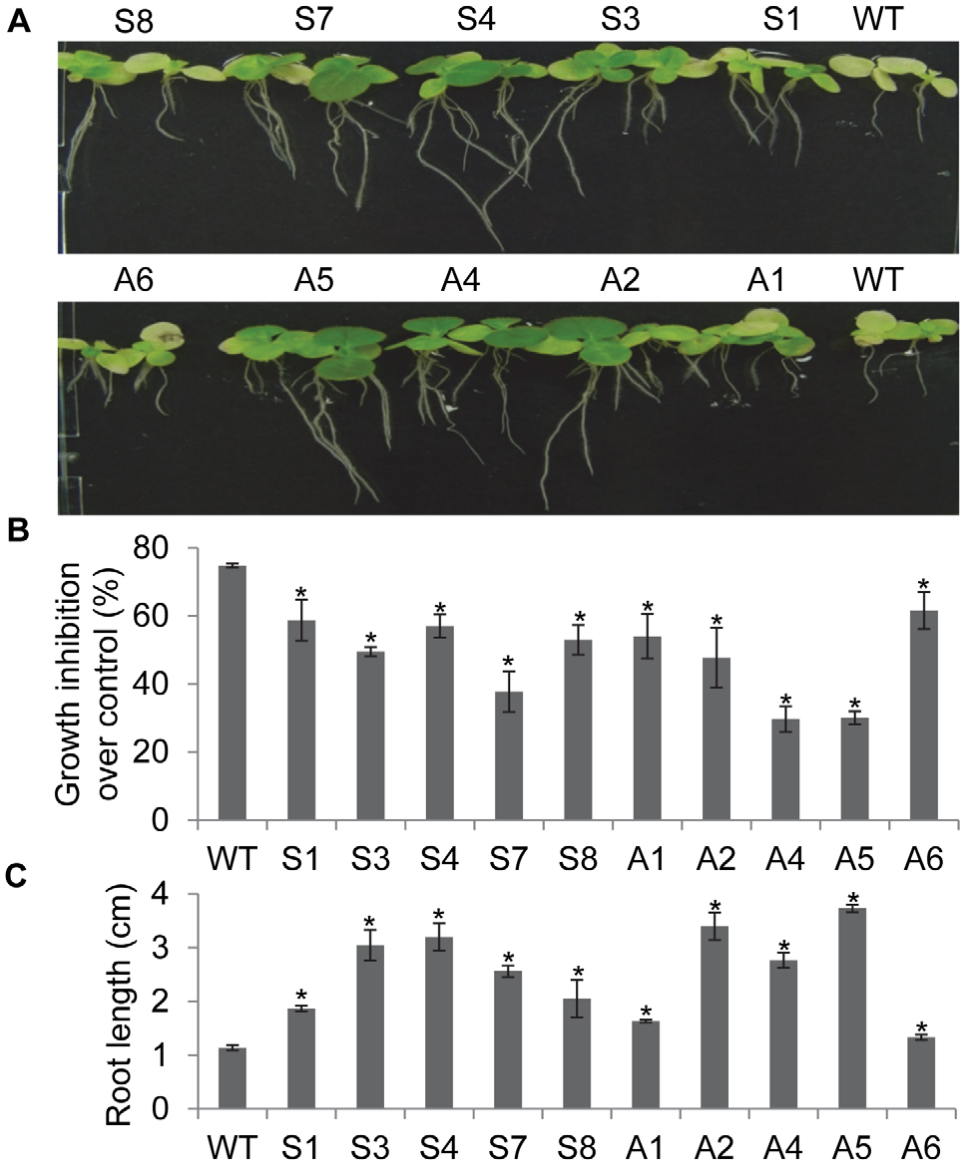
Short-term salt stress response of *EcNAC1* transgenic tobacco plants. 15-day-old T_1_ seedlings selected on hygromycin were inter-planted with wild-type seedlings on MS medium supplemented with 200 mM NaCl, and the observations were taken after seven days. (A) Phenotype, (B) Reduction in fresh weights over control and (C) Root elongation of transgenic tobacco plants under salt stress. Each bar value represents the mean ± sd (n = 6) of triplicate experiments (student’s t test; *P<0.05 versus wild-type).

Under short-term salt stress, the transgenic seedlings maintained better growth and showed significantly less reduction in fresh weight than wild-type (Figure 4A & B). Root elongation was also significantly more in all the transgenic seedlings compared to that of wild-type even under high salt stress (Figure 4C). Similarly, under long-term exposure to different NaCl concentrations the reduction in growth was less in transgenic plants and they maintained better leaf growth and chlorophyll content than the wild-type plants even at 200 mM NaCl (Figure S6A & B), suggesting that the overexpression of *EcNAC1* in tobacco confers a high degree of tolerance to salinity stress.

### *EcNAC1* Tobacco Plants Show Improved Tolerance to MV-induced Oxidative Stress

Under short-term stress, transgenic plants survived and maintained growth at 5 µM MV for seven days when all the wild-type plants were completely bleached (Figure S7A). Transgenic plants accumulated distinctly higher fresh weight (Figure S7B) and showed significantly better root growth under stress compared to wild-type (Figure S7C). Even under long-term exposure transgenic plants expressing *EcNAC1* constitutively, survived whereas the wild-type plants were not able to grow and died within 10 days (data not shown). However, none of the plants survived at higher concentration of MV (10 µM).

### *EcNAC1* Transgenic Plants Show Better Water-deficit Stress Tolerance and Recovery

**Figure 5:**
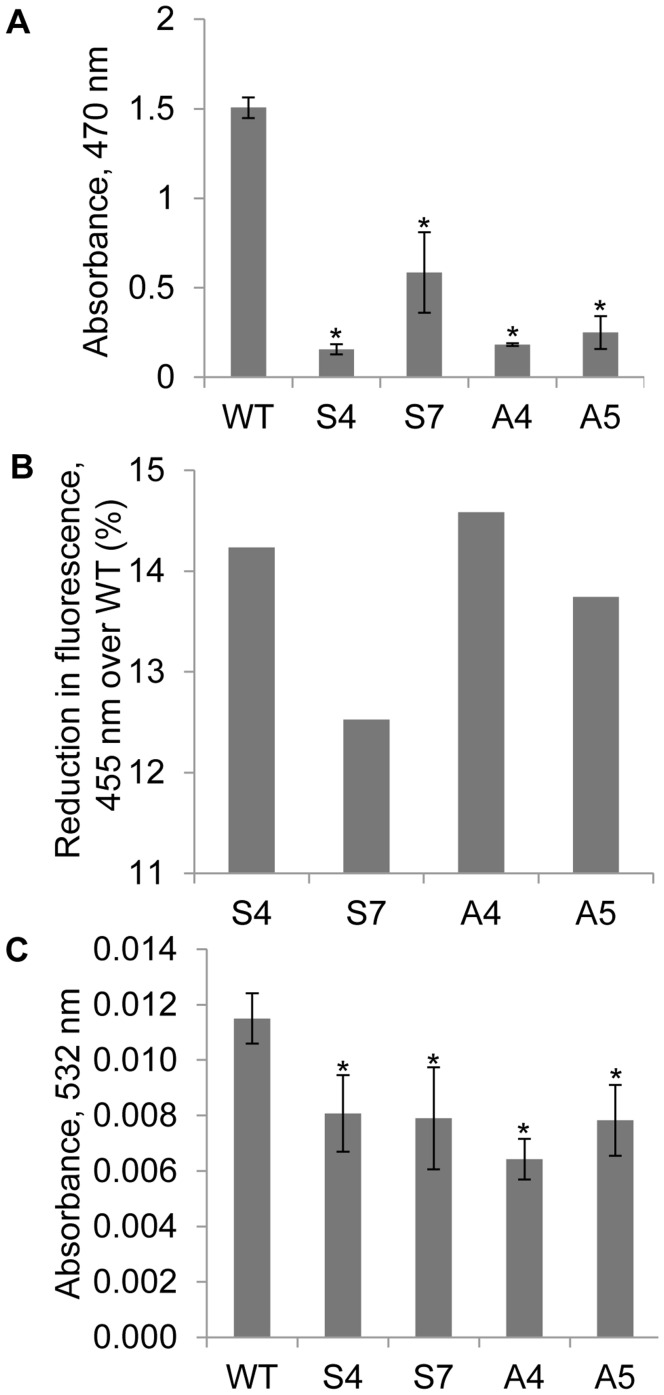
ROS scavenging activity of transgenic plants under water-deficit stress. 15-day-old T_1_ seedlings were transferred to pots and allowed to establish for 20 days. Water-deficit stress was imposed by gravimetric approach and plants were maintained at 35% FC for 10 days. (A) O_2_.^−^, (B) H_2_O_2_, and (C).OH radical content were quantified by XTT, scopoletin, and 2-deoxy-D-ribose assay, respectively. Each bar value represents the mean ± sd of triplicate experiments (student’s t test; *P<0.05 versus wild-type).

**Figure 6:**
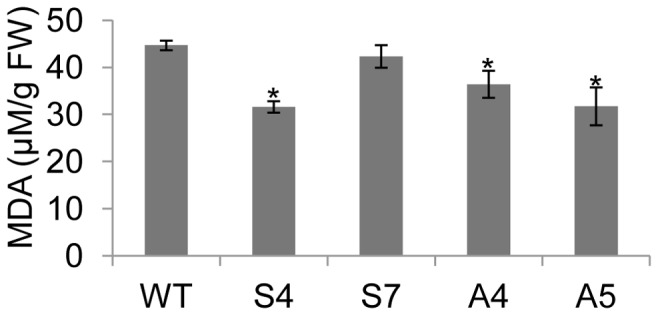
Lipid peroxidation in wild-type and transgenic plants under water-deficit stress. In water-deficit stressed plants MDA accumulation was measured in terms of μM/g fresh weight (FW). Each bar value represents the mean ± sd of triplicate experiments (student’s t test; *P<0.05 versus wild-type).

Gradual water-deficit stress was imposed till the soil FC reaches to 35% and subsequently the plants were maintained at same FC for 10 days. To test the free radical scavenging activity of *EcNAC1* overexpressed plants under water-deficit stress, the ROS (O_2_.^−^, H_2_O_2_, and OH radical) levels were quantified. Transgenic plants showed 5–7 fold lower O_2_.^−^ content over wild-type plants (Figure 5A). The H_2_O_2_ content was 12.5–14.5% lower in transgenic plants compared to wild-type plants (Figure 5B). Even the levels of OH radicals were significantly lower in transgenic plants suggesting higher free radical scavenging activity under water-deficit conditions (Figure 5C). The level of lipid peroxidation in both transgenic and wild-type plants was estimated in terms of amount of MDA accumulated. All transgenic lines tested, except line S7, showed significantly lower MDA than the wild-type under water-deficit stress (Figure 6). The lower MDA levels in transgenic plants suggest that the *EcNAC1* tobacco plants have less lipid peroxidation.

**Figure 7:**
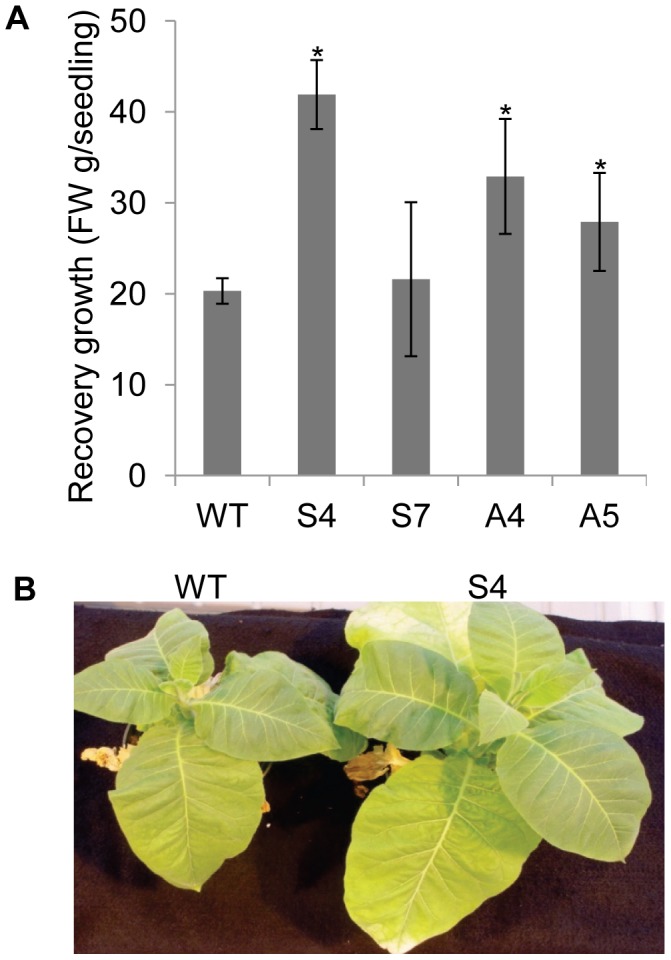
Recovery response of transgenic plants subjected to water-deficit stress. At the end of water-deficit stress period plants were re-watered and observations were taken after 15-days. (A) Recovery growth and (B) Phenotype of wild-type and transgenic plants. Each bar value represents the mean ± sd of triplicate experiments (student’s t test; *P<0.05 versus wild-type).

The recovery response of the stressed transgenic and wild-type plants was studied 15-days after re-watering. All the transgenic plants tested, except line S7, recovered faster and accumulated significantly higher fresh weight when compared to wild-type (Figure 7A). Representative recovery phenotype of S4 and wild-type plants is shown in Figure 7B. These results showed relevance of *EcNAC1* gene under water-deficit stress.

### Overexpression of *EcNAC1* Activates a Few Known NAC Target genes in Tobacco

To further understand whether the stress-tolerance observed in *EcNAC1* overexpressing tobacco plants was due to transcriptional up-regulation of downstream stress-responsive genes, we analysed the relative transcript levels of several stress-responsive genes. The transgenic line S4 was chosen to study the possible regulation of selected genes by *EcNAC1,* as this line showed less growth inhibition even at 300 mM NaCl, better root growth under 300 mM mannitol, superior ROS scavenging activity and also lower MDA under water-deficit stress. Apart from that, this line also survived under long-term exposure to MV-induced oxidative stress. Expression of 14 target genes of *SNAC1,* [17], was analyzed by RT-PCR. The transcripts of genes under study were observed in both transgenic and wild-type tobacco plants and the levels of *WUSCHEL-related homeobox 13 (HB-13), MYB-CC type transcription factor (MYB), protein phosphatase 2C (PP2C), serine-threonine protein kinase (STPK), NADPH-cytP450 oxidoreductase (CytOR), Rop subfamily GTPase (ROP)*, and *ATP-binding subunit, ERD1* were more in the transgenic line when compared to wild-type as shown by the increase in relative density of the RT-PCR products (Figure S8). Based on these results we suggest that *EcNAC1* can induce a few stress associated genes there by bringing in improved stress-tolerance in transgenic tobacco plants.

## Discussion

Stress-responsive transcription factors that might regulate unique signaling pathways are relevant in bringing the desired levels of tolerance to plants. NAC family transcription factors, specific to plants, have been implicated in plant developmental programs [55], [56], defense [16], [57] and abiotic stress responses [19], [20], [58]. Only a few stress-responsive NAC proteins have been characterized [59] for their relevance in imparting tolerance to abiotic stresses. From this context, characterizing other *NAC* family genes or the stress-responsive homologs from stress adapted species provides greater insight about this unique group of transcription factors.

Several studies have shown that stress adapted species have structurally efficient genes and regulatory pathways which impart stress-tolerance. For example, overexpression of Na^+^/H^+^ antiporter gene, *ApNhaP*, from a halo-tolerant Cyanobacterium has drastically improved the salt-tolerance of freshwater Cyanobacterium [34]. To identify stress-responsive transcription factors from a stress adapted plant species, finger millet, subtracted stress cDNA library was constructed and a gene encoding NAC protein was identified. Sequence analysis of *EcNAC1* gene isolated from the library suggested that *EcNAC1* is closely related to a few known stress-responsive rice genes that encode NAC proteins. Previously, Fang et al. [12] have systematically analyzed protein sequences of 140 putative *NAC* or *NAC*-like genes in rice and classified them into five groups (I–V). Based on trait specificity, stress-related *NAC* genes fell into the group III stress-responsive *NAC* genes. Phylogenetic analysis of deduced amino acid sequence of EcNAC1 along with rice NAC proteins and also the closest accessions of maize and sorghum showed that this gene actually represents the group III, suggesting its stress-responsive nature. MEME-based prediction of putative motifs among this cluster showed that N-terminal regions were conserved in parallel with NAC domain structure and EcNAC1 appear to have unique motif 6 in the C-terminal region. In a recent study, it was shown that overexpression of *PINO1,* from a halophytic wild rice *Porteresia coarctata*, in rice and tobacco has substantially improved salt tolerance compared to its domesticated rice homolog *RINO1* which lacks a unique stretch of 37 amino acids [35]. The same group also identified *PcIMT1* gene, from *Porteresia coarctata*, which is absent in domesticated rice and overexpression of this gene has resulted in accumulation of pinitol under salt stress in rice [60] suggesting stress adapted species might have novel candidate genes or unique mechanisms which could help genetic engineering of susceptible species for improved stress-tolerance. Expression analysis of *EcNAC1* in our studies also confirms its stress-responsiveness. *EcNAC1* was induced in finger millet seedlings/plants exposed to water-deficit and salinity stress. Among several *NAC* genes identified in *Arabidopsis* and rice, only a few were shown to be stress-responsive [15], [17]–[20], [22], [58], [61], [62] and are grouped under stress-responsive *NAC* genes. The promoter regions of some of these genes have DRE and ABRE sequences [58], [63]. The promoter characterization of *EcNAC1* which has highest homology with rice *SNAC1* could help in understanding the pathway through which the gene is regulated.

The major focus in this study was to identify genes from drought adapted crop, finger millet, and study whether genes from tolerant species can impart better stress-tolerance in a susceptible species. We used tobacco as susceptible species and tested the relevance of *EcNAC1* for stress-tolerance. As tobacco was used as system to transform *EcNAC1*, the stress-responsive nature of endogenous *NAC* gene was also analysed to understand the regulation of related NAC genes in tobacco. The closest homolog of a partial fragment was identified from TIGR database (TIGR index# TC5942) and designated as *NtNAC*. qRT-PCR analysis showed more than 10 and 5 fold higher expression of the EST over control under dehydration and salinity, respectively, suggesting the stress induction of related *NAC* genes in tobacco (Figure S9). There are contrasting reports on the effect of constitutive overproduction of regulatory molecules, such as transcription factors, on growth under normal condition [24], [64]–[67]. However, overexpression of *EcNAC1* in tobacco plants did not affect growth under normal condition. Similar results were observed in overexpression studies of other transcription factors. Oh et al. [68] reported that the transgenic plants expressing *CBF3* (*C-repeat binding factor 3*) under *Ubiquitin1* promoter exhibited no visible phenotypic alterations in rice. These reports and our present result suggest that the growth abnormalities observed in many transgenic plants constitutively expressing the regulatory genes is not a universal phenomenon.

Most of the *EcNAC1* overexpressing transgenic lines showed increased tolerance to osmotic and salinity stress. The growth rates of transgenic lines on simulated osmotic stress by PEG were remarkably higher than wild-type plants under short-term stress. Such a response was also observed under long-term stress experiments. Transgenic plants expressing NAC transcription factors including *SNAC1* [17], *ONAC063* [19], and *OsNAC5* [58] have shown osmotic stress-tolerance at various stages. Overexpression of *SNAC1* not only improved drought resistance at vegetative stage in transgenic rice but also improved yield under field stress [17]. Overexpression of *SNAC2* also effectively improved PEG-induced osmotic stress-tolerance in rice seedlings [18]. *Arabidopsis* seedlings overexpressing *ONAC063* showed higher germination rates under mannitol-induced osmotic stress [19]. Conversely, overexpression of seven other *NAC* genes, whose expression is induced by exogenous ABA treatment, drought or high salinity [69], did not impart salt tolerance in *Arabidopsis* [23], suggesting all stress-induced genes may not give tolerant phenotype. In our study, *EcNAC1* was induced by salinity and we also observed superior salt tolerance of many *EcNAC1* transgenics.

Other notable observation is that substantial superior root growth of *EcNAC1* transgenic lines compared to the improved shoot growth. Recently a few studies have shown the role of NAC proteins in root growth thereby improving salt stress-tolerance. He et al. [70] have suggested the involvement of *NAC* genes in improving *Arabidopsis* root growth under salinity stress. Overexpression of *AtNAC1* and *AtNAC2* resulted in altered lateral root development. In addition, the auxin responsive genes *AIR3* (*Auxin Induced in Root cultures 3*) and *DBP* (*DNA-Binding Protein*) were identified in a screen for downstream targets of *NAC1* [14]. Overexpression of *GmNAC20* has promoted lateral root formation in *Arabidopsis* under salt stress [71]. However, there is no direct evidence to support the involvement of NAC transcription factors in root growth under osmotic/dehydration and oxidative stress. We observed that under PEG and mannitol-induced osmotic stress, high salinity as well as oxidative stress there was significant increase in the root growth of *EcNAC1* transgenic plants when compared to wild-type tobacco plants. Transgenic plants showed not only improved root growth but also better lateral root formation. Understanding the phytohormone signaling pathway in *NAC* overexpressing plants may provide further insight in this direction.

By *in-vitro* studies using transgenic *EcNAC1* seedlings, we provided evidences that *EcNAC1* imparts tolerance to MV-induced oxidative stress. One of the major factors when plants are exposed to stress under high light is the generation of cytotoxic compounds like ROS [72]. The levels of these cytotoxic compounds in pot grown transgenic plants subjected to gradual water-deficit stress under natural high light condition was relatively less. These results are further substantiated by lower lipid peroxidation in transgenic plants as shown by lower levels of MDA. Thus, it is conceivable that the ROS-scavenging and membrane-protecting properties of *EcNAC1* accounts, at least partially, for the enhanced tolerance to oxidative stress in transgenic tobacco plants. In rice *OsNAC6* overexpression plants, several biotic and abiotic responsive genes were up-regulated. A transient trans-activation assay also demonstrated the expression of peroxidase in *OsNAC6* transformants. Further, in a recent study by Balazadeh et al. [73], it was demonstrated that the up-regulation of several downstream stress-responsive genes having consensus *cis*-elements [RCGTR(4–5n)RYACGCAA; R = A or G; Y = C or T] is regulated by NAC transcription factors. Among them, many are oxidative stress associated genes. This signifies the roles of a few NAC transcription factors in regulation of oxidative stress-tolerance.

We observed the expression pattern of closest tobacco homologs of a few *SNAC1* target genes [17]; presuming *EcNAC1* might also have common target genes to regulate because of its 57% identity at protein level with *SNAC1*. There was up-regulation of a few stress-responsive genes such as *HB-13, MYB, PP2C*, *STPK*, *CytOR*, *ROP* and *ERD1.* The involvement of some of these genes in stress-tolerance is well studied (HD proteins [74]; MYB related transcription factors [66]; *PP2C* [75]; serine-threonine protein kinase [76]; Rop subfamily GTPase [77]; *ERD1* [62]). NAC transcription factor has been shown previously to activate several downstream functional genes involved in stress adaptation [17], [20], [58]. Even though different NAC transcription factors interact with core *cis*-acting sequences in the promoters of target genes but the actual set of target genes for each paralog vary in different plants. Both *cis*-acting DNA sequences and transacting transcription factor amino acid sequences have co-evolved to regulate the coordinated expression of several stress adaptive genes. Similarly, the architecture of promoter sequences within these stress-related genes are not simply collinear, they are multifunctional and modular, and interact with multiple independently regulated transcription factors and display a transcriptional complexity to cope up with the cross-talk in response to various environmental stresses. Taken together these results indicate that transgenic overexpression of *EcNAC1* in tobacco positively correlates with stress-tolerance.

In conclusion, this study shows that *EcNAC1* is stress-inducible and several known stress-responsive genes are up-regulated in tobacco transgenic plants expressing *EcNAC1* thus conferring tolerance to various abiotic stresses including dehydration, salinity and oxidative stress. Our work suggests the possibility of engineering abiotic stress-tolerance in a crop plant by overexpressing *EcNAC1* gene from a heterologous plant species.

## Supporting Information

Figure S1 **Phenotype of finger millet plants grown at different levels of water-deficit stress.** Gradual stress was imposed on 25-day-old pot grown plants following gravimetric approach. Stress was applied for a set of plants over a period of 5 days to reach 80% FC, likewise 8 days to reach 60% and 10 days to reach 35% FC. Samples were collected on the same day. Rain-out shelter was used to protect the plants from adverse weather; otherwise plants were exposed to natural vapor pressure deficit. Photographs were taken at the end of stress period.
(TIF)

Figure S2 **PCR analysis of putative T_0_ tobacco transformants expressing** ***EcNAC1*** **under** ***4xABRE*** **stress-inducible promoter.** Genomic DNA was isolated from tobacco plants transformed with *4xABRE::EcNAC1:NOS* and PCR reactions were performed with (A) *HPTII* forward and reverse, (B) Gene-specific forward and NOS terminator reverse, (C) Gene-specific forward and reverse and (D) Promoter forward and gene-specific reverse primers to confirm the integration. M - marker; P - plasmid.
(TIF)

Figure S3 **PCR analysis of putative T_0_ tobacco transformants expressing** ***EcNAC1*** **under** ***CaMV35S*** **constitutive promoter.** Genomic DNA was isolated from tobacco plants transformed with *CaMV35S::EcNAC1:NOS* and PCR reactions were performed with (A) *HPTII* forward and reverse, (B) Gene-specific forward and NOS terminator reverse, (C) Gene specific forward and reverse and (D) Promoter forward and gene-specific reverse primers to confirm the integration. M - marker; P - plasmid.
(TIF)

Figure S4 **RT-PCR analysis of wild-type and** ***EcNAC1*** **expressing tobacco plants.** A. Expression of the transgene analyzed by RT-PCR. B. The corresponding increase in the relative density of bands over wild-type. M - marker; P - plasmid.
(TIF)

Figure S5 **Long-term osmotic stress response of transgenic tobacco plants expressing** ***EcNAC1*** **.** 15-day-old T_1_ transgenic seedlings selected on hygromycin were transferred to MS medium supplemented with 100, 200 and 300 mM mannitol and observations were taken after 30-days. (A) Phenotype, (B) Root fresh weight of wild-type and transgenic plants. Each bar value represents the mean ± sd (n = 6) of triplicate experiments (student’s t test; *P<0.05 versus wild-type).
(TIF)

Figure S6 **Long-term salt stress response of transgenic tobacco plants expressing** ***EcNAC1*** **.** 15-day-old T_1_ seedlings selected on hygromycin were transferred to MS medium supplemented with 100, 200 and 300 mM of NaCl and observations were taken after 30-days. (A) Phenotype, (B) Growth inhibition of wild-type and transgenic plants. Each bar value represents the mean ± sd (n = 6) of triplicate experiments (student’s t test; * P<0.05 versus wild-type).
(TIF)

Figure S7 **MV-induced short-term oxidative stress response of** ***EcNAC1*** **transgenic tobacco plants.** 15-day-old T_1_ seedlings selected on hygromycin were inter-planted with wild-type seedlings on MS medium amended with 5 μM MV and the observations were taken after seven days. (A) Phenotype, (B) Fresh weights and, (C) Root elongation of transgenic tobacco plants. Each bar value represents the mean ± sd (n = 12) of triplicate experiments (student’s t test; *P<0.05 versus wild-type).
(TIF)

Figure S8 **Expression pattern of known NAC target genes in wild-type and S4 transgenic tobacco plants.** Transcript levels of various target genes of *SNAC1* were determined by RT-PCR analysis. The *EF1α* gene was amplified as control. Bars in the right panel show the change in relative density of RT-PCR products when compared to wild-type. *HB13-WUSCHEL-related homeobox 13; MYB-MYB-CC type transcription factor; NAM10-NAM-like protein 10; PP2C-protein phosphatase 2C; STPK-serine-threonine protein kinase; CytOR-NADPH-cytP450 oxidoreductase; ROP-Rop subfamily GTPase; ERD1-ATP-binding subunit-early responsive to dehydration; NHT-sodium/dicarboxylate co-transporter like; Chap21-chaperonin 21 precursor; NT-nitrate transporter; HVA22-similar to AtHVA22 like protein; GT-similar to probable glycosyltransferase; and PO_4_R-phosphate-responsive 1 family protein.*
(TIF)

Figure S9 **qRT-PCR analysis of** ***NtNAC*** **induction during dehydration and salt stress in tobacco.** qRT-PCR was performed with the total RNA isolated from leaf tissue as described in material methods. The *Elongation factor 1α* gene was used as normaliser.
(TIF)

Table S1 **Primers used in the study.**
(DOCX)

Table S2 **Tobacco homologs of rice** ***SNAC1*** **target genes used in the expression analysis.**
(DOCX)

Table S3 **Motifs identified in EcNAC1 deduced amino acid sequence.**
(DOCX)
