# Supplementary figures and images for: Expression of a Finger Millet Transcription Factor, EcNAC1, in Tobacco Confers Abiotic Stress-Tolerance
Source: PLoS One. 2012 Jul 11;7(7):e40397. doi: 10.1371/journal.pone.0040397 (PMC3394802; doi:10.1371/journal.pone.0040397)

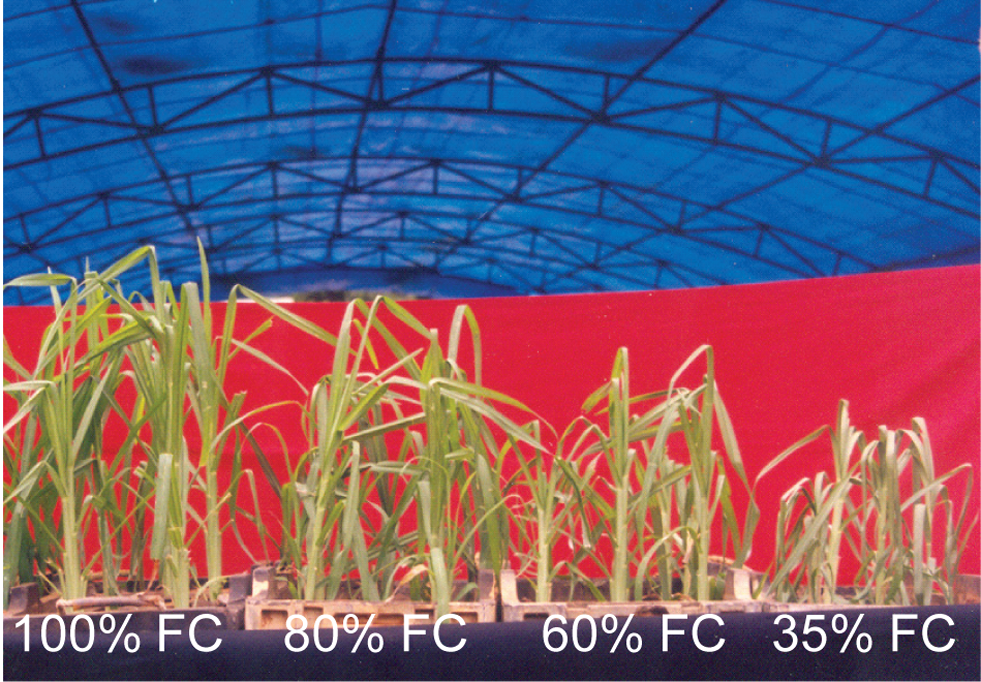

Supplement: Figure S1 — Phenotype of finger millet plants grown at different levels of water-deficit stress. Gradual stress was imposed on 25-day-old pot grown plants following gravimetric approach. Stress was applied for a set of plants over a period of 5 days to reach 80% FC, likewise 8 days to reach 60% and 10 days to reach 35% FC. Samples were collected on the same day. Rain-out shelter was used to protect the plants from adverse weather; otherwise plants were exposed to natural vapor pressure deficit. Photographs were taken at the end of stress period. (TIF) [file pone.0040397.s001.tif]

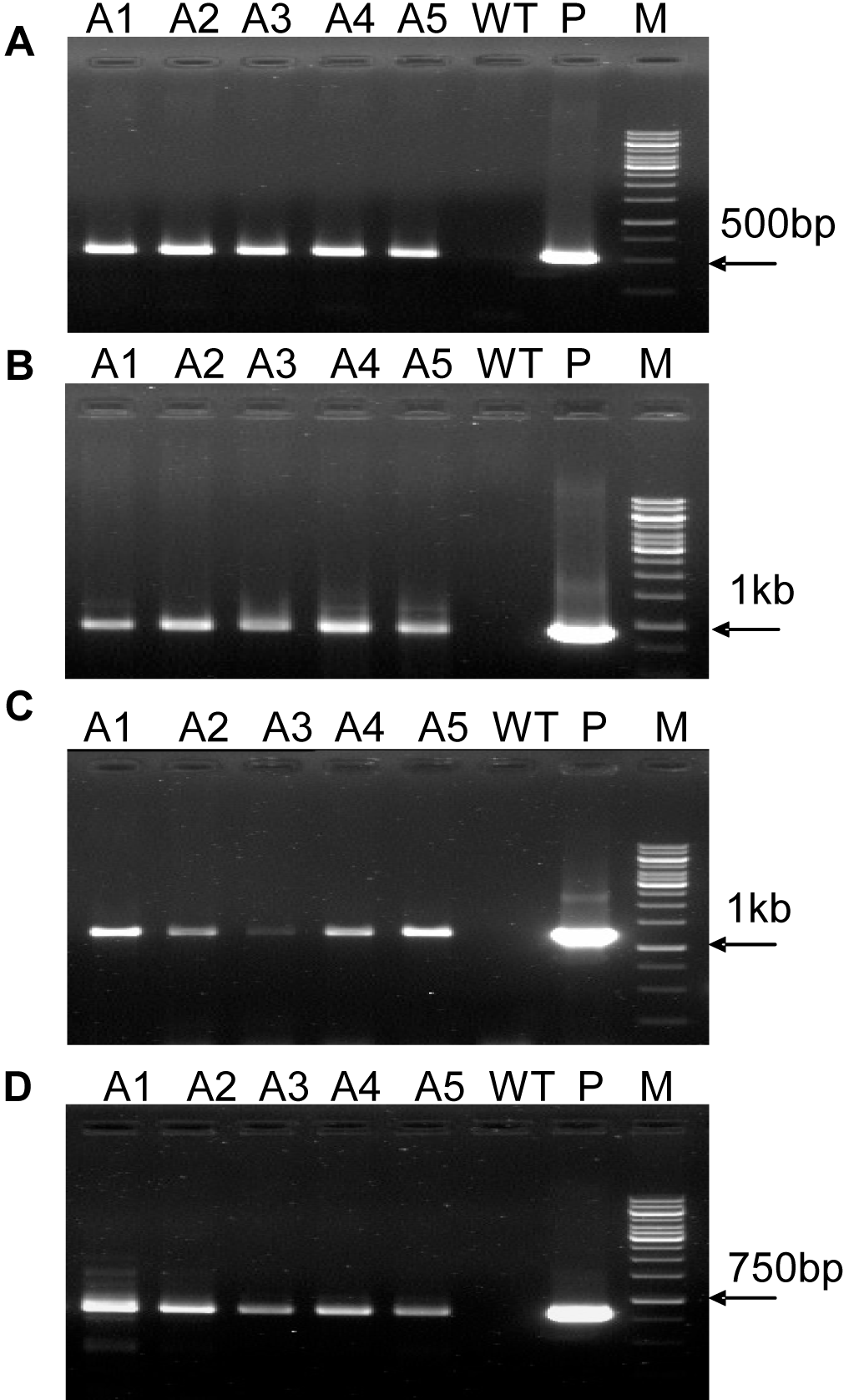

Supplement: Figure S2 — PCR analysis of putative T0 tobacco transformants expressing EcNAC1 under 4xABRE stress-inducible promoter. Genomic DNA was isolated from tobacco plants transformed with 4xABRE::EcNAC1:NOS and PCR reactions were performed with (A) HPTII forward and reverse, (B) Gene-specific forward and NOS terminator reverse, (C) Gene-specific forward and reverse and (D) Promoter forward and gene-specific reverse primers to confirm the integration. M - marker; P - plasmid. (TIF) [file pone.0040397.s002.tif]

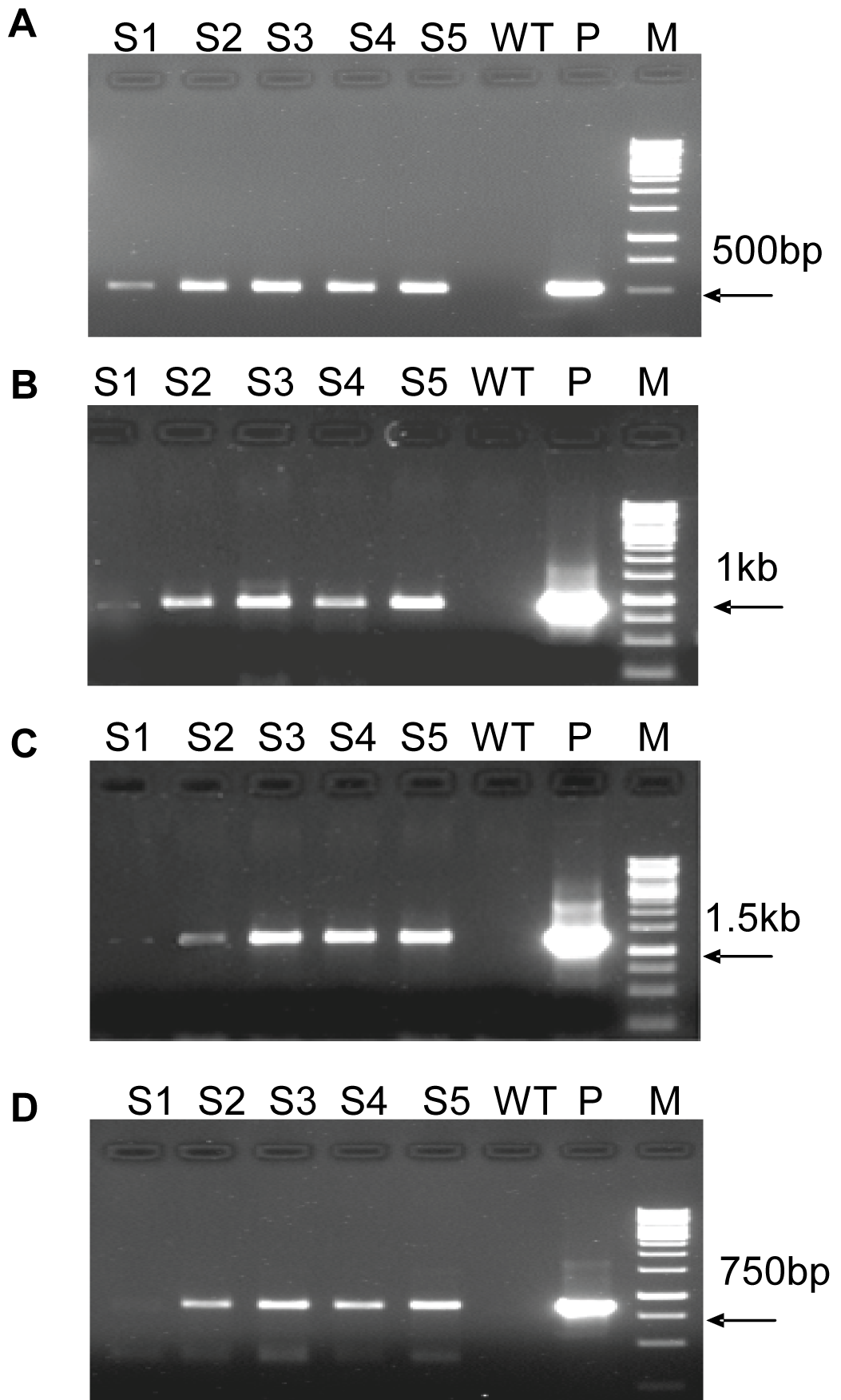

Supplement: Figure S3 — PCR analysis of putative T0 tobacco transformants expressing EcNAC1 under CaMV35S constitutive promoter. Genomic DNA was isolated from tobacco plants transformed with CaMV35S::EcNAC1:NOS and PCR reactions were performed with (A) HPTII forward and reverse, (B) Gene-specific forward and NOS terminator reverse, (C) Gene specific forward and reverse and (D) Promoter forward and gene-specific reverse primers to confirm the integration. M - marker; P - plasmid. (TIF) [file pone.0040397.s003.tif]

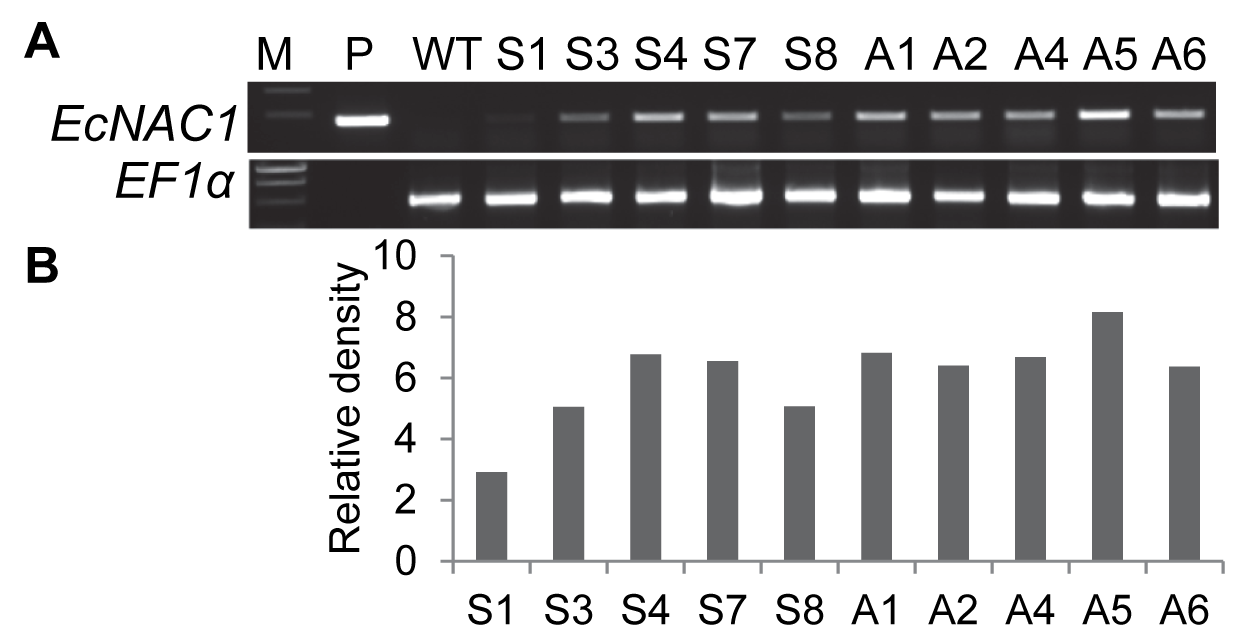

Supplement: Figure S4 — RT-PCR analysis of wild-type and EcNAC1 expressing tobacco plants. A. Expression of the transgene analyzed by RT-PCR. B. The corresponding increase in the relative density of bands over wild-type. M - marker; P - plasmid. (TIF) [file pone.0040397.s004.tif]

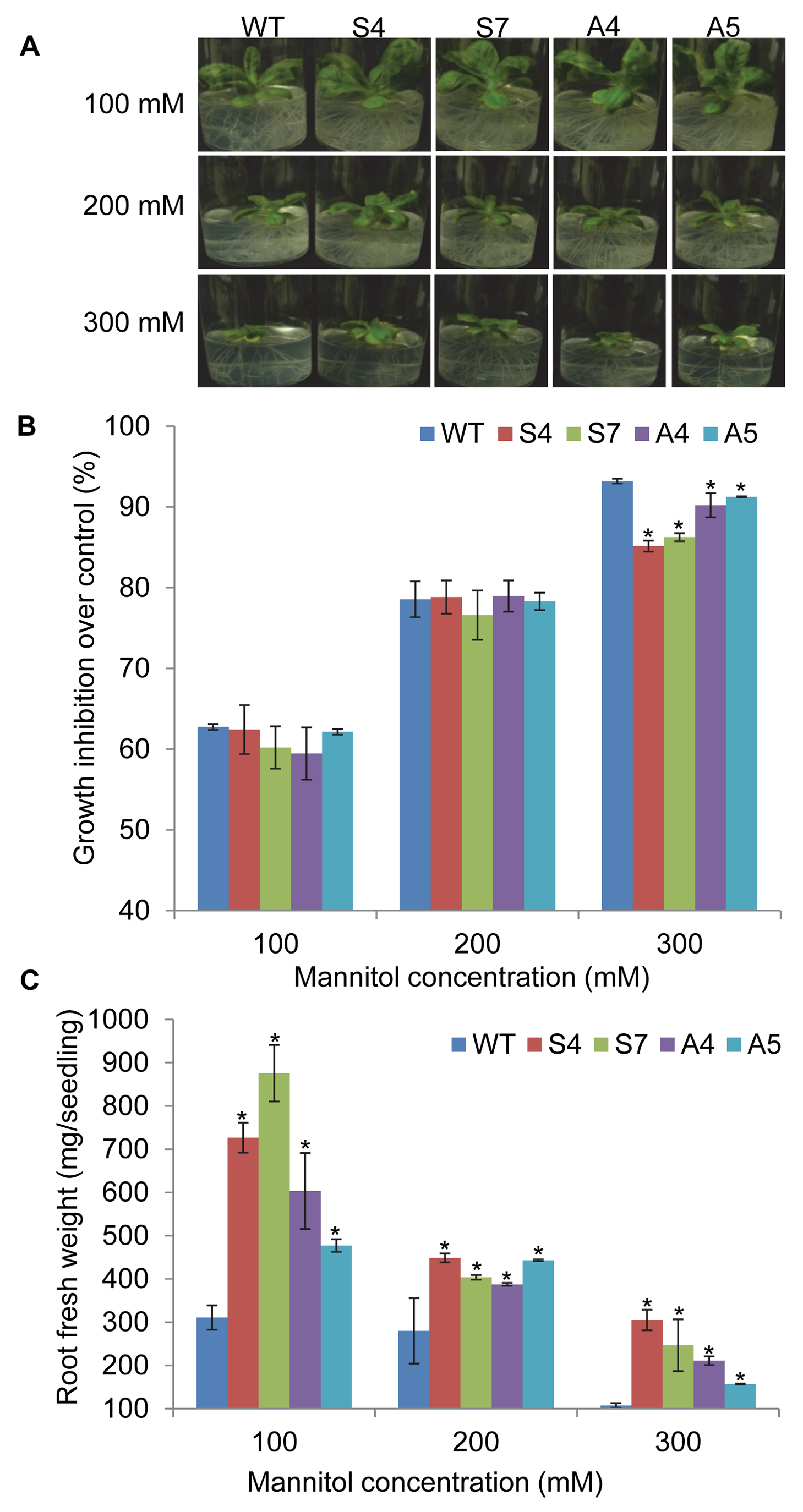

Supplement: Figure S5 — Long-term osmotic stress response of transgenic tobacco plants expressing EcNAC1 . 15-day-old T1 transgenic seedlings selected on hygromycin were transferred to MS medium supplemented with 100, 200 and 300 mM mannitol and observations were taken after 30-days. (A) Phenotype, (B) Root fresh weight of wild-type and transgenic plants. Each bar value represents the mean ± sd (n = 6) of triplicate experiments (student’s t test; *P<0.05 versus wild-type). (TIF) [file pone.0040397.s005.tif]

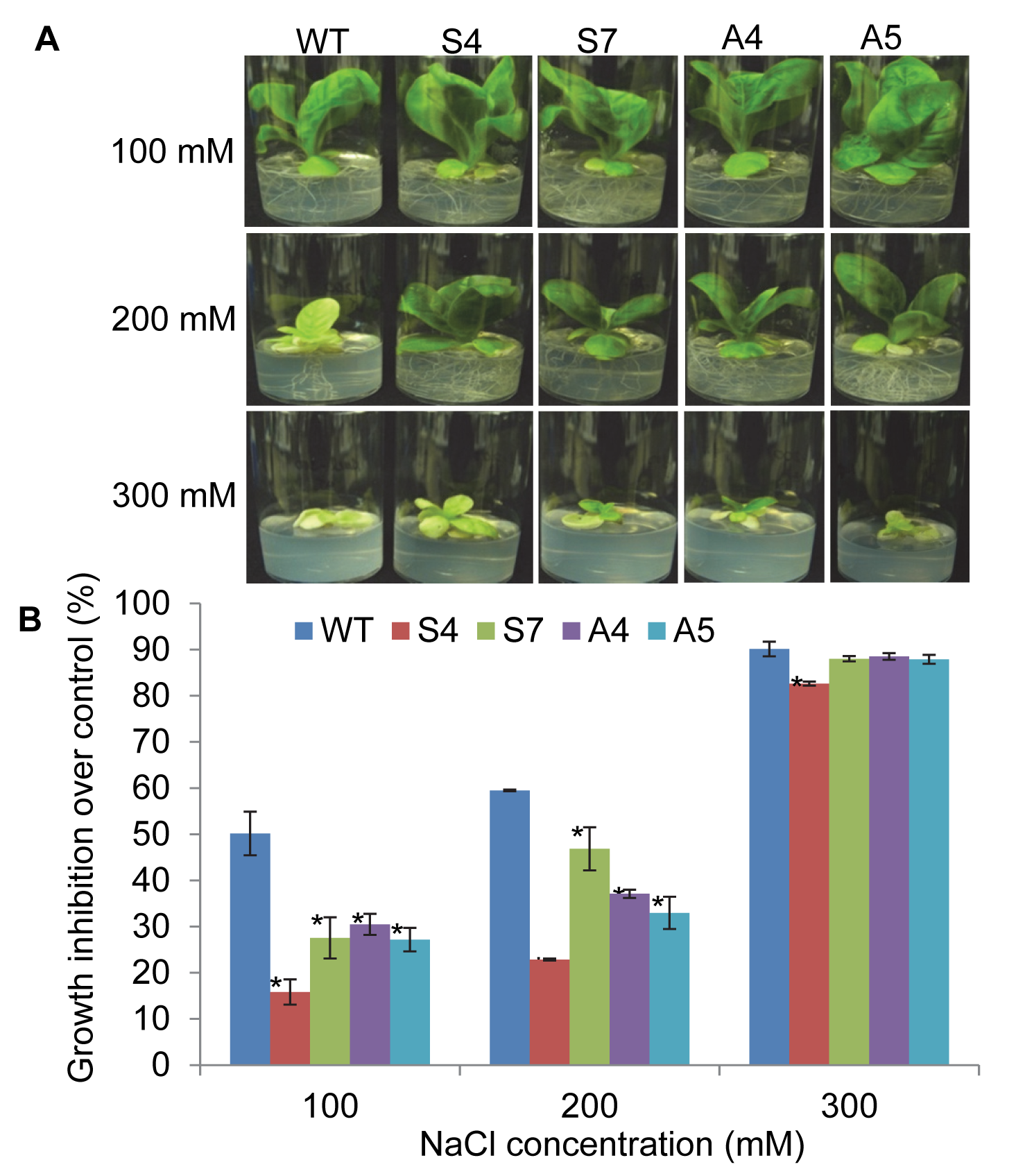

Supplement: Figure S6 — Long-term salt stress response of transgenic tobacco plants expressing EcNAC1 . 15-day-old T1 seedlings selected on hygromycin were transferred to MS medium supplemented with 100, 200 and 300 mM of NaCl and observations were taken after 30-days. (A) Phenotype, (B) Growth inhibition of wild-type and transgenic plants. Each bar value represents the mean ± sd (n = 6) of triplicate experiments (student’s t test; * P<0.05 versus wild-type). (TIF) [file pone.0040397.s006.tif]

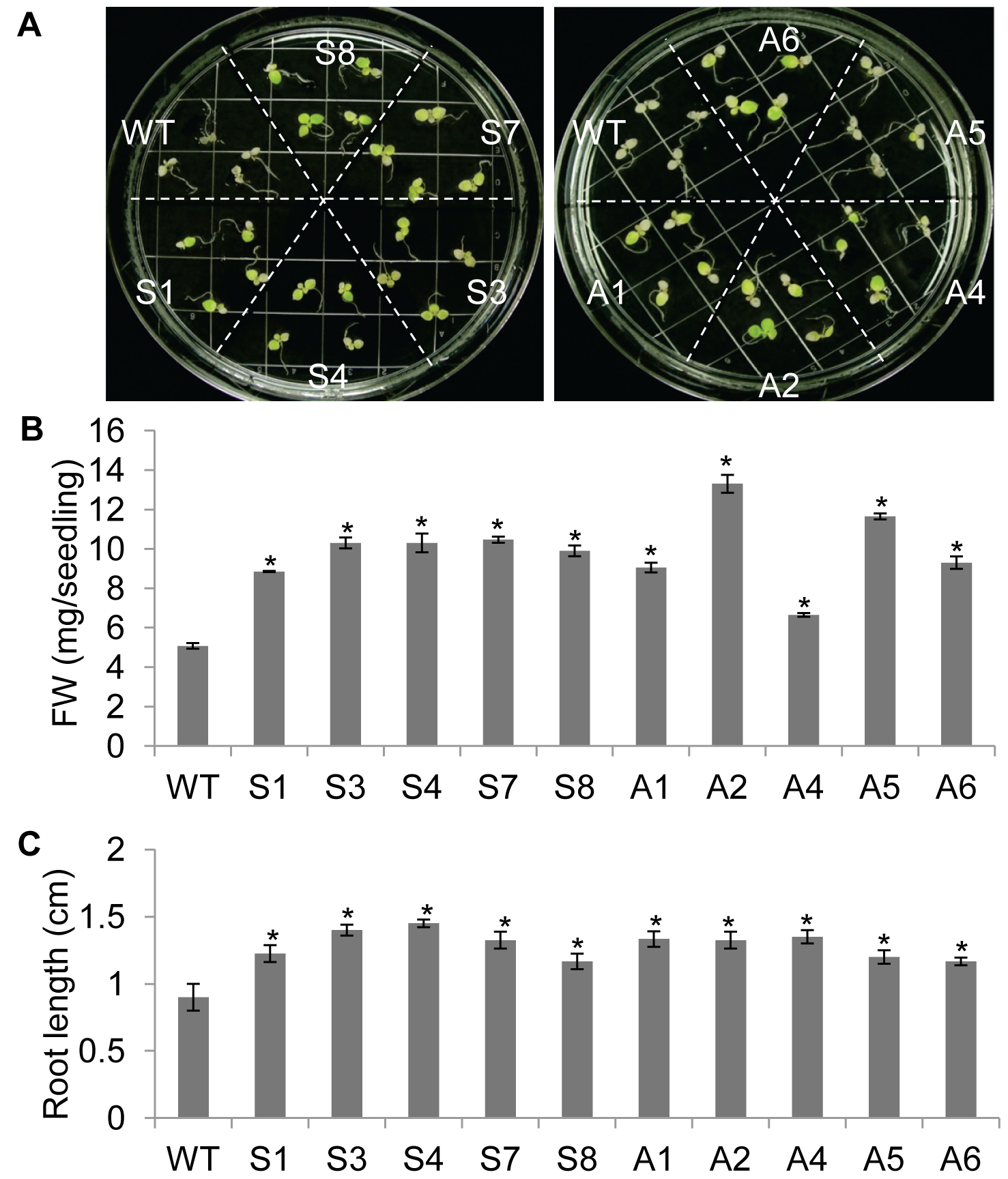

Supplement: Figure S7 — MV-induced short-term oxidative stress response of EcNAC1 transgenic tobacco plants. 15-day-old T1 seedlings selected on hygromycin were inter-planted with wild-type seedlings on MS medium amended with 5 μM MV and the observations were taken after seven days. (A) Phenotype, (B) Fresh weights and, (C) Root elongation of transgenic tobacco plants. Each bar value represents the mean ± sd (n = 12) of triplicate experiments (student’s t test; *P<0.05 versus wild-type). (TIF) [file pone.0040397.s007.tif]

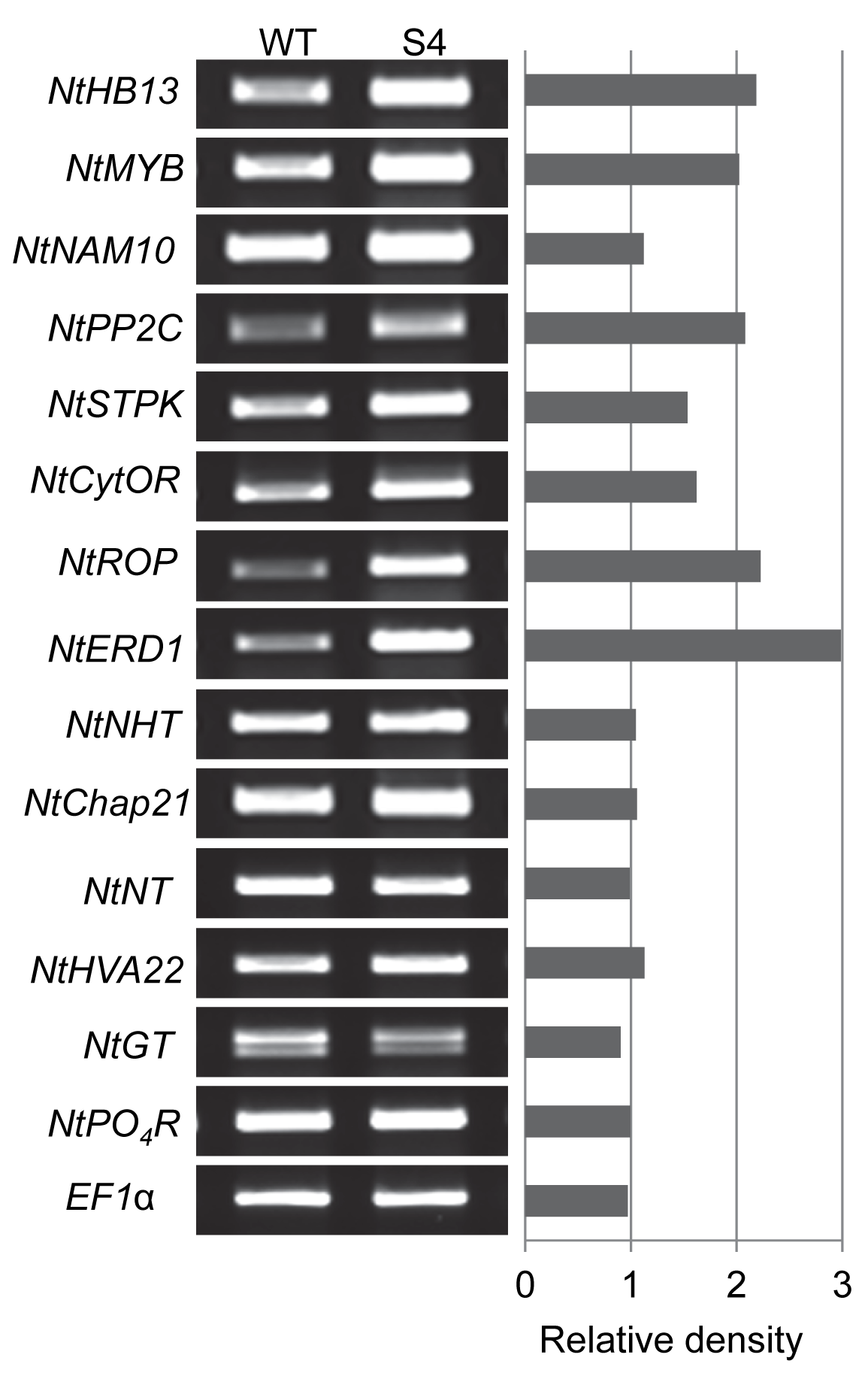

Supplement: Figure S8 — Expression pattern of known NAC target genes in wild-type and S4 transgenic tobacco plants. Transcript levels of various target genes of SNAC1 were determined by RT-PCR analysis. The EF1α gene was amplified as control. Bars in the right panel show the change in relative density of RT-PCR products when compared to wild-type. HB13-WUSCHEL-related homeobox 13; MYB-MYB-CC type transcription factor; NAM10-NAM-like protein 10; PP2C-protein phosphatase 2C; STPK-serine-threonine protein kinase; CytOR-NADPH-cytP450 oxidoreductase; ROP-Rop subfamily GTPase; ERD1-ATP-binding subunit-early responsive to dehydration; NHT-sodium/dicarboxylate co-transporter like; Chap21-chaperonin 21 precursor; NT-nitrate transporter; HVA22-similar to AtHVA22 like protein; GT-similar to probable glycosyltransferase; and PO4R-phosphate-responsive 1 family protein. (TIF) [file pone.0040397.s008.tif]

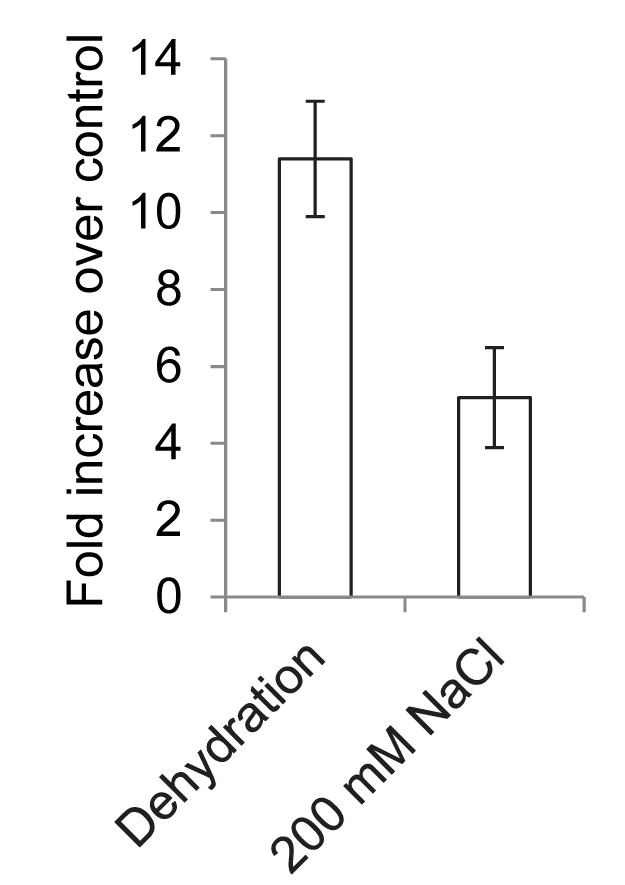

Supplement: Figure S9 — qRT-PCR analysis of NtNAC induction during dehydration and salt stress in tobacco. qRT-PCR was performed with the total RNA isolated from leaf tissue as described in material methods. The Elongation factor 1α gene was used as normaliser. (TIF) [file pone.0040397.s009.tif]
