# Supplementary material for: Expression of a Finger Millet Transcription Factor, EcNAC1, in Tobacco Confers Abiotic Stress-Tolerance
Source: PLoS One. 2012 Jul 11;7(7):e40397. doi: 10.1371/journal.pone.0040397 (PMC3394802; doi:10.1371/journal.pone.0040397)
Supplement: Table S1 — Primers used in the study. (DOCX) [file pone.0040397.s010.docx]

**Table S1. Primers used in the study.**

| A. Amplification of *EcNAC1* coding region | |
| --- | --- |
| *EcNAC1* ORF | 5′-ATGACCATGGGAGGAGGGATGATG-3′ |
|  | 5′-TTACAGAGGTCGCAGCTGCAGTCC-3′ |

| B. Expression analysis of *EcNAC1* | |
| --- | --- |
| *EcNAC1* | 5′-CGAGTACCGTCTTGCTGATGCC-3′ |
|  | 5′-CGAGTGCGAGTGCGAGTTCC-3′ |
| *Actin* | 5’-TCCATAATGAAGTGTGATGT-3’ |
|  | 5’-GGACCTGACTCGTCATACTC-3’ |

| C. Expression analysis of known *NAC* target genes | |
| --- | --- |
| *NtHB13* | 5′-AACATCAGCTGGCCACAGAATCAG-3′ |
|  | 5′-TTAGGTGATTCGACCTCTGTCTCC-3′ |
| *NtMYB* | 5′-GGTGGCAACACAATGGAGCCAATA-3′ |
|  | 5′-GCGGATGAACCATCCAAACTGGAA-3′ |
| *NtNAM10* | 5′-GTGGCAGATGACAGGAATGAGT-3′ |
|  | 5′-AACCTTTGCTTGGTCACTAGCCGA-3′ |
| *NtPP2C* | 5′-AGCCGATGCATACAGCCATACAGA-3′ |
|  | 5′-CAAACGCACGGGAAACAGCAAGTA-3′ |
| *NtSTPKF* | 5′-GTAGGCTACCTTACTCGGCAAACA-3′ |
|  | 5′-ATCTCAAGCTCAGCCCACCATAGA-3′ |
| *Nt CytOR* | 5′-AATCTTGCATCACCGGCTGGAAAG-3′ |
|  | 5′-TCATCCATGTTGAGCAGACACCCT-3′ |
| *NtAROP* | 5′-TGTCAATGGGAGCACTGTCAACCT-3′ |
|  | 5′-TAGTAATTGGCACAGCACCGGGAT-3′ |
| *NtERD1* | 5′-GCCATGCATGAAGTGATCTTGGCA-3′ |
|  | 5′-ACAAATGGCTGCAACAGCCTCATC-3′ |
| *NtNHT* | 5′-TGCTTTGTGGGCTACACTTTGCTC-3′ |
|  | 5′-TCTCCTGGCTGCTTCTTGTTTGGA-3′ |
| *NtChap-21* | 5′-AAACCTCAAGGAGGTGAGGTGGTT-3′ |
|  | 5′-TCCTTTGCTGCCTCAGTCAACAGT-3′ |
| *NtNT* | 5′-ATTCCGTTTCTTGGACAAGGCAGC-3′ |
|  | 5′-AAGAAGAGTGTCAATGAGGCCGGA-3′ |
| *NtHVA22* | 5′-ACTAGAGGGCTTGTGCTGGTCTTT-3′ |
|  | 5′-GTCTCATGCCTTAACACTACGGGT-3′ |
| *NtGT* | 5′-TGAATGAGGATCGGACGTTGTGGA-3′ |
|  | 5′-TGAGGCGTAACAGTGTGCTTCTCT-3′ |
| *NtPO_4_R* | 5′-ATATGAGGGCTCGTCGGAGTTTGT-3′ |
|  | 5′-TTTCTCTTCGCCGAGGTGGACATT-3′ |
| *ELF 1α* | 5′-ATCAGGACAGCACAGTCAGCTT-3′ |
|  | 5′-ACGTGAGCGTGGTATCACCATT-3′ |

| D. Expression analysis of *NtNAC* | |
| --- | --- |
| *NtNAC* | 5′-ATGTGAAGCCGGAGATTCTG-3′ |
|  | 5′-TGAGCGATTGGGAGAAAACT-3′′ |

| E. Other primers used in the PCR analysis of *EcNAC1* transgenic plants. | |
| --- | --- |
| *HPTII* | 5′-AGCTGCGCCGATGGTTTCTACAA-3’ |
|  | 5′-ATCGCCTCGCTCCAGTCAATG-3’ |
| *NOSt* reverse | 5′-CGATCTAGTAACATAGATGACACCGCGC-3’ |
| *CaMV35S* promoter forward | 5′-CATGGAGTCAAAGATTCAAATAGAGGACC-3’ |
| *4xABRE* promoter forward | 5′-ATGACGCACAATCCCACTATCCTT-3’ |
