# Supplementary material for: Expression of a Finger Millet Transcription Factor, EcNAC1, in Tobacco Confers Abiotic Stress-Tolerance
Source: PLoS One. 2012 Jul 11;7(7):e40397. doi: 10.1371/journal.pone.0040397 (PMC3394802; doi:10.1371/journal.pone.0040397)
Supplement: Table S2 — Tobacco homologs of rice SNAC1 target genes used in the expression analysis. (DOCX) [file pone.0040397.s011.docx]

**Table S2. Tobacco homologs of rice *SNAC1* target genes used in the expression analysis.**

| **GenBank accession # for rice *SNAC1* target genes** | **Annotation** | **TIGR gene index for tobacco homologs** | **Annotation** | **Homology (%)** |
| --- | --- | --- | --- | --- |
| AK059910 | Homeobox-leucine zipper protein (*HB-2*) | TC4378 | WUSCHEL-related homeobox 13 | 67 |
| AK103241 | MYB family transcription factor | TC8157 | MYB-CC type transfactor | 66 |
| AK065989 | NAM protein | TC5942 | NAM-like ptotein 10 | 64 |
| AK066016 | Protein phosphatase 2C putative | TC11278 | Protein phosphatase 2C, partial | 75 |
| AK067959 | Leucine-rich repeat transmembrane protein kinase | TC11770 | Serine-threonine protein kinase | 60 |
| AK068915 | NADPH-ferrihemoprotein reductase | TC7720 | NADPH-cytP450 oxidoreductase | 72 |
| AK107382 | Rac-like GTP-binding protein | TC7058 | Rop subfamily GTPase | 59 |
| AK068727 | ATP-binding subunit (ClpD), (*OsERD1*) | TC48611 | ATP-dependent Clp protease ATP-binding subunit clpA homolog CD4B, chloroplast precursor | 69 |
| AK072183 | Sodium/dicarboxylate cotransporter | TC7595 | Sodium/dicarboxylate cotransporter like | 61 |
| AK060474 | 20-kDa chaperonin | TC7352 | Chaperonin 21 precursor | 71 |
| AK099405 | Proton-dependent oligopeptide transport protein | TC3963 | Nitrate transporter | 52 |
| AK058393 | ABA-responsive HVA22 family protein | TC5661 | Similar to *AtHVA22* like protein | 65 |
| AK066736 | Glycosyl transferase family 8 protein | TC5762 | Similar to probable glycosyltransferase | 62 |
| AK068474 | Phosphate-responsive 1 family protein | TC7610 | Similar to At5g51550/K17N15_10 | 63 |
