# Supplementary material for: Expression of a Finger Millet Transcription Factor, EcNAC1, in Tobacco Confers Abiotic Stress-Tolerance
Source: PLoS One. 2012 Jul 11;7(7):e40397. doi: 10.1371/journal.pone.0040397 (PMC3394802; doi:10.1371/journal.pone.0040397)
Supplement: Table S3 — Motifs identified in EcNAC1 deduced amino acid sequence. (DOCX) [file pone.0040397.s012.docx]

**Table S3. Motifs identified in EcNAC1 deduced amino acid sequence.**

| **Motif no.** | **E-value** | **Annotation of motif** | **Conserved amino acids of motif** |
| --- | --- | --- | --- |
| 1 | 5.7E-20 | NAM | RERDAEAELNLPPGFRFHPTDDELVEHYLCRKAAGQRLPVPIIAEVDLYKFDPWDLPDRALFGTGEWYFF |
| 2 | 1.00E-13 | NAM | ATGADKPVAPKGRTLGIKKALVFYAGKAPRGVKTDWIMHEYRLADAGRAAAAKKGSLRLDD |
| 3 | 2.30E-09 | NAM | TPRDRKYPNGSRPNRAAGNGYWKATGADKPVAPKGRTLGIK |
| 4 | 2.80E-93 | Unknown | KKGSLRLDDWVLCRLYNKKNEWEKMQMKKG |
| 5 | 2.10E-91 | Unknown | DLPDRALFGTGEWYFFTPRDRKYPNGSRPN |
| 7 | 1.30E-07 | Unknown | SDLFVDLSYDDIQGMYSGLDMLPP |
| 9 | 2.40E-06 | Unknown | AGRAAAAKKGSLRLDDWVLCRLYNKK |

*The motif numbers correspond to the numbers in Figure 1B.
